# Residential Radon in Manizales, Colombia: Results of a Pilot Study

**DOI:** 10.3390/ijerph18031228

**Published:** 2021-01-29

**Authors:** Alexandra Giraldo-Osorio, Alberto Ruano-Ravina, Mónica Pérez-Ríos, Leonor Varela-Lema, Juan Miguel Barros-Dios, Nelson Enrique Arias-Ortiz

**Affiliations:** 1 Department of Preventive Medicine and Public Health, University of Santiago de Compostela, 15782 Santiago de Compostela, Spain; alexandra.giraldo.osorio@usc.es (A.G.-O.); monica.perez.rios@usc.es (M.P.-R.); leonor.varela@usc.es (L.V.-L.); juanm.barros@usc.es (J.M.B.-D.); 2 Grupo de Investigación Promoción de la Salud y Prevención de la Enfermedad (GIPSPE), Departamento de Salud Pública, Universidad de Caldas, Manizales 170002, Colombia; nelson.arias@ucaldas.edu.co; 3 Scholarship Holder of Fundación Carolina (C.2020), 28071 Madrid, Spain; 4 Consortium for Biomedical Research in Epidemiology & Public Health (CIBER en Epidemiología and Salud Pública/CIBERESP), 15782 Santiago de Compostela, Spain; 5 Health Research Institute of Santiago de Compostela (Instituto de Investigación Sanitaria de Santiago de Compostela—IDIS), 15706 Santiago de Compostela, Spain

**Keywords:** radon, lung cancer, dwelling, cross-sectional studies, natural radiation, public health, Colombia

## Abstract

Radon is a colorless, odorless, and tasteless noble gas, causally related with the onset of lung cancer. We aimed to describe the distribution of radon exposure in the municipality of Manizales, Colombia, in order to estimate the population’s exposure and establish the percentage of dwellings that surpass reference levels. A cross-sectional study representing all geographical areas was carried out by measuring indoor radon concentrations. Participants answered a short questionnaire. Alpha-track type radon detectors were installed in all residences for six months. The detectors were subsequently processed at the Galician Radon Laboratory, an accredited laboratory at the University of Santiago de Compostela. A total of 202 homes were measured. Seventy-seven percent of the sampled houses were three stories high, their median age was 30 years, and half were inhabited by three people or fewer. For most dwellings, the building materials of walls and flooring were brick and covered cement, respectively. Results showed a geometric mean of radon concentration of 8.5 Bq/m^3^ and a maximum value of 50 Bq/m^3^. No statistically significant differences were found either between the geometric mean of the dwelling’s site, the height at which detectors were placed inside the home, or the wall and flooring materials, or between mean ^222^Rn concentrations in rural and urban areas. No dwelling surpassed the ^222^Rn reference level established by the WHO. This study shows that residential radon levels in Manizales, Colombia, seem to be low, though a more in-depth approach should be carried out. Despite these results, it is essential to create a national radon program and establish a radon concentration reference level for Colombia in line with international recommendations.

## 1. Introduction

Radon is a decay product of uranium, which is present in rocks and soils present on the Earth’s surface. It is colorless, odorless, and tasteless. Its most relevant isotope, epidemiologically speaking, is ^222^Rn, as it accounts for 80% of all isotopes [1]. Radon is a radioactive element and, in its decay, produces alpha ionizing radiation. In itself, ^222^Rn does not pose a health risk, given that its half-life is only 3.8 days, and therefore a small percentage of inhaled gas will decay in the respiratory tract. However, radon’s radioactive decay yields other products with a very short half-life, ^218^Polonium and ^214^Polonium, which also produce alpha radiation. These, when inhaled for a prolonged length of time, damage the pulmonary cell lining, causing genetic alterations, which in turn may lead to the onset of lung cancer [2,3]. Though radon is currently recognized as only causing lung cancer, there is some evidence pointing to a link with COPD, brain cancer, stomach cancer, or esophageal cancer [4,5,6,7,8,9,10].

Radon has been declared carcinogenic in humans by the International Agency for Research on Cancer (IARC) [11]. It is the second risk factor for lung cancer after cigarette smoking and the first in never-smokers [2,12,13]; the risk of developing lung cancer rises 16% per 100 Bq/m^3^ increase in indoor radon concentrations [2]. It is also well-known that exposure to residential radon is the greatest source of natural radiation [14]. The Environment Protection Agency (EPA) in the United States estimates that radon is responsible for approximately 20,000 lung cancer deaths annually. In order to address this issue, the World Health Organization (WHO) has established a recommended radon concentration under 100 Bq/m^3^, with a maximum level of 300 Bq/m^3^ [1] (2.7 pCi/L and 8.1 pCi/L, respectively; 1 pCi/L is equal to 37 Bq/m^3^). Despite the strong recommendations of international organizations such as WHO [13], the US EPA [15], and the International Commission of Radiological Protection (ICRP) [16], based on studies with thousands of patients and studies with relevant sample sizes [3,17] stating that there is no threshold effect, there are some authors still claiming that there could be such a threshold [18,19,20]. Of note, most countries and international organizations (European Union, ICRP, Canada) have lowered the action levels in recent years, clearly disregarding such a hypothetical threshold (reviewed in [21]).

In Colombia, around 52,000 people die every year of lung cancer, which is second in terms of mortality, only surpassed by stomach cancer [22]. In 2016, lung cancer age adjusted mortality rate in Manizales was 23.4 in men and 12.8 in women, which amounts to nearly double that of the whole country [23]. Part of this risk excess in Manizales could be explained by greater exposure to tobacco (prevalence of consumption in 2019 and its last month was 17.4 and 14.7%, respectively, compared to 12.1% and 9.8% in Colombia) [24]. Likewise, there could be some occupational exposure to lung carcinogens, given that Manizales is a city with an industrial tradition, mainly in metallurgy, manufacture of electronic devices, and chemical and plastic/rubber industries [25]. Nevertheless, the prevalence of radon exposure in Colombia, both in the workplace and in dwellings, is unknown. There is only one study for the municipality of Manizales, specifically in its southeast area, that included measurements taken from locations surrounding active volcanoes and from the air in 18 buildings (among houses, kindergartens, schools, and hospitals); the method of detection used consisted of electrets, and the total duration of the measurement was 15 days [26,27]. There are no other studies in Colombia and only a few in South America describing indoor radon concentrations (reviewed in [28]).

The aim of this study was to describe the distribution of exposure to residential radon in the municipality of Manizales, Colombia, in order to estimate the population’s exposure and the percentage of dwellings that surpass reference levels.

## 2. Materials and Methods

### 2.1. Design and Setting

A cross-sectional study was carried out to measure indoor radon concentrations in the municipality of Manizales. Manizales is the capital of Caldas, a department in central western Colombia situated at an average altitude of 2150 m.a.s.l. in the central Andes. The city center is laid down on a low-mountain humid forest ecosystem [29]. Wind patterns in Manizales are low speed (average 0.6 m/s), which hampers the dispersion of atmospheric contaminants [30]. The city’s climate is warm–humid, with wide ranging variations in daytime temperature (12–24 °C), atmospheric pressure (785–795 hPa), and relative humidity (50–100%) [30]. Rains follow a bimodal pattern (March–May; September–November) with dry periods in between, preferential orientation toward the NW and SW and are inversely proportional to altitude [31]. Manizales has a population of 434,403, according to the National Administrative Department of Statistics (*DANE*) [32]. Regarding its political–administrative division, its urban area is divided onto 12 communes and its rural area into 7 jurisdictions. The local Land Registry listed a total of 137,585 buildings (9359 rural and 128,226 urban constructions) at 13 April 2013 [33].

### 2.2. Sampling

The objective was to obtain radon concentration values representative of the population; therefore, radon detectors were placed to represent populated areas, with more detectors in place in those most densely populated sectors. All departmental jurisdictions (rural areas) and communes (urban areas) in the municipality of Manizales were included. In each jurisdiction and commune, sampling was organized in terms of convenience. Thus, in rural areas (population 30,000 inhabitants approximately), the criterion was using one detector per 1000 inhabitants in each jurisdiction (at least 30 detectors for the whole rural area) and an extra device per each extra 1000 subjects. In urban areas, we applied a criterion of a minimum of 10 radon detectors with a number of inhabitants equal or under 30,000 and, again, an additional device per each 1000 extra inhabitants. We did not consider the number of stories of the building to place the radon detectors, meaning that in urban areas we measured a first or a fifth floor indistinctly. Dwellings were selected independent of their height. Inhabitants of selected homes were contacted, and the closest dwelling was chosen in situ if any were uninhabited or if the owner was unwilling to participate. Homes inhabited all year round by a resident over 18 years old and willing to take part were included, and we excluded homes whose inhabitants were planning to move within three months after our visit. Participants were informed that their geolocalization and anonymity would be kept and were asked to sign an informed consent form. An identification form was developed (short questionnaire) as used in previous studies [34], which was adapted to Colombian types of dwelling and building materials [35]. Through it, information on the age of the house, building materials used indoors (flooring and walls), existence of a cellar, and number of stories was obtained.

### 2.3. Residential Radon Measurements

We used alpha-track (CR-39, manufactured by Radosys Kft® located in Budapest, Hungary) passive radon detectors, which employ a microscopic recounting of alpha particles to provide an annual estimation of indoor radon concentrations [36]. Two researchers personally placed the detectors in the participants’ homes, in the main bedroom, following the instructions of other studies that had previously used this type of detector [34]. The same researchers (AGO and NEAO) retrieved the detectors, taking note if these detectors had been moved and checking the number of days the detectors had been exposed. All participants underwent a follow-up to check the correct placement of the detector during the length of the study (January–June 2020). Finally, the devices were hermetically sealed and shipped by urgent air mail to the Galician Radon Laboratory in Galicia, Spain, where they were analyzed. The Galicia Radon Laboratory at the University of Santiago de Compostela is one of the three Spanish labs accredited by the (Spanish) National Accreditation Entity to carry out radon measurements in the air [34]. This certification recognizes that radon measurements provided are reliable and reproducible since the laboratory follows the standards proposed by the rule *“Una Norma Española-European Norm”* (*UNE-EN*) ISO 11665-4:2020 to measure radon in air. This procedure includes a series of quality check steps (i.e., blank detectors or detectors with a predefined concentration). Each participant was eventually sent a report with the results.

### 2.4. Statistical Analysis

We used a descriptive analysis for both rural and urban areas, comprising minimum and maximum concentrations, geometric mean, geometric standard deviation, and 95% confidence intervals; median, 25th, and 75th percentiles; and average number of dwellings with radon concentration levels over 100 Bq/m^3^. We used this value to display low, medium, and high radon concentration areas. Bivariate analyses were performed to associate indoor radon exposure with urban or rural setting and also to associate radon concentration with floor of the dwelling (Wilcoxon test). The analysis was performed using Microsoft Excel 2018 (Microsoft Corporation, Albuquerque, NM, USA) and Stata/IC 16.1 (StataCorp College Station, TX, USA).

## 3. Results

**Figure 1:**
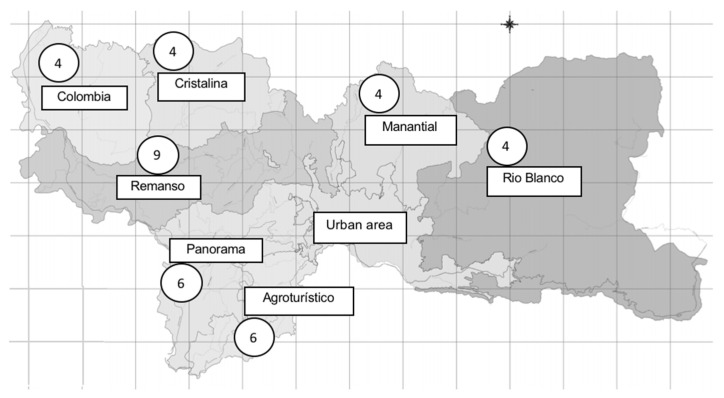
Number of detectors distributed in each jurisdiction of the rural area of the municipality of Manizales, Colombia. January to June 2020. Source: Alcaldía de Manizales, Secretaría de Planeación, Gestión de información Geográfica.

**Figure 2:**
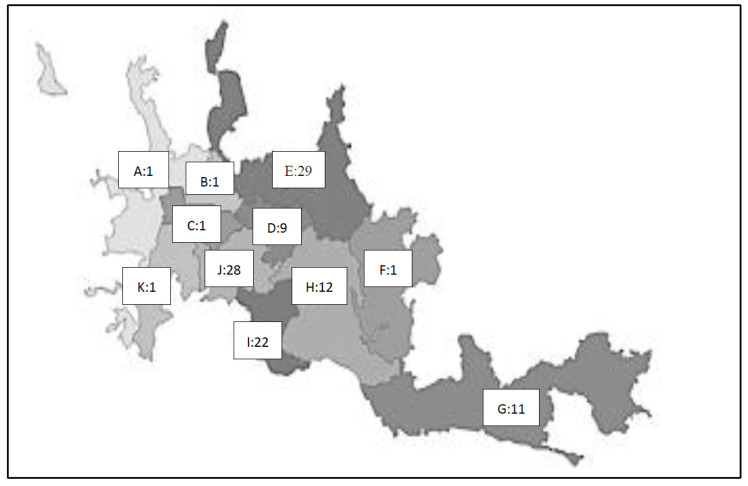
Number of detectors distributed in each commune of the urban area of the municipality of Manizales, Colombia. January to June 2020. The communes that appear on the map are: Atardeceres (**A**), San José (**B**), Cumanday (**C**), La Estación (**D**), Ciudadela del Norte y Comuna 12 (**E**), Ecoturístico Cerro de Oro (**F**), Tesorito (**G**), Palogrande (**H**), Universitaria (**I**), La Fuente (**J**), and La Macarena (**K**). Source: Alcaldía de Manizales, Secretaría de Planeación, Gestión de información Geográfica.

Two hundred and ten detectors were distributed throughout the different urban (173) and rural selected homes (37) (Figure 1 and Figure 2). All participants were offered information on the process, and receptivity was high; the rate of acceptance in the study was over 95%. Rejected cases were asked to take part in the in situ assessment of residents in the closest nearby dwellings. There was a total of eight (3.8%) devices lost due to diverse causes.

Measurements were obtained from homes in rural areas (83.2%) and urban areas (16.8%). All devices were placed in the main bedrooms. Eighty-three percent of dwellings were houses and 17% were apartments. Median age of dwellings was 30 (interquatilic range = 29 years); half were inhabited by 3 people or fewer. None had air conditioning.

**Figure 3:**
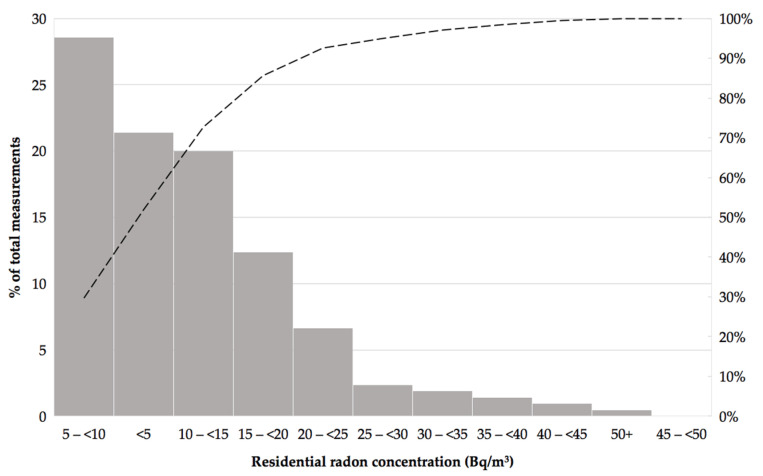
Residential ^222^Rn concentrations in Manizales, Colombia. January to June 2020.

Median residential radon concentration in Manizales was 9 Bq/m^3^, and the 25th and 75th percentiles were 5 Bq/m^3^ and 16 Bq/m^3^, respectively. The maximum value was 50 Bq/m^3^, and the minimum was below the detection limit of our reading procedures. In this case the reading was assigned to 0 Bq/m^3^, after subtracting the background radiation of each batch of detectors used. Indoor radon data followed a log-normal distribution with a geometric mean of 8.5 Bq/m^3^ and a geometric standard deviation of 2.4 Bq/m^3^. Approximately 90% of measurements showed radon levels <25 Bq/m^3^ (Figure 3).

**Figure 4:**
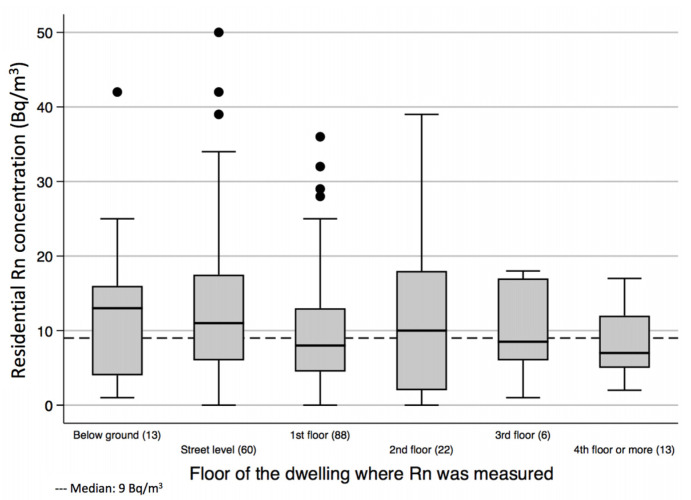
Residential ^222^Rn concentrations according to the height of the measurement site. Manizales, Colombia. January to June 2020.

Seventy-seven percent of the sampled houses were three stories high, and 30% of bedrooms were at street level, 44% were in the second story, and 11% in the third; 6.4% were below ground level. Median radon concentration was similar regarding the height at which the detector was placed in the dwelling, as shown in Figure 4, although radon concentrations were slightly higher below ground.

Table 1 shows mean (geometric) radon concentrations for different dwelling characteristics. In most dwellings, the building materials of walls and flooring were brick and covered cement, respectively. There were no significant statistical differences in median ^222^Rn by location (urban/rural) of dwellings (median test = 0.249; *p* = 0.618), by measurement site height (median test = 10.976; *p* = 0.052), or by materials of walls (median test = 1.471; *p* = 0.479). Material of floors showed a difference (median test = 6.811; *p* = 0.033).

Radon concentrations were >30 Bq/m^3^ for 4.9% of dwellings, 30% of which were in urban areas. No significant statistical differences were found regarding the age of the homes when comparing dwellings with concentrations over 30 Bq/m^3^ and under 30 Bq/m^3^ (Wilcoxon test = 0.092; *p* = 0.9290).

We asked participants about tobacco consumption and found a prevalence of 10.0% (*n* = 21) current smokers and 21.8% (*n* = 44) former smokers.

## 4. Discussion

Our aim was to describe the distribution of residential radon in the municipality of Manizales, Colombia, in order to estimate the population’s exposure and the percentage of dwellings that surpass reference levels. The results show that residential ^222^Rn concentration levels in Manizales were low since no dwelling surpassed 100 Bq/m^3^. This research is pioneering in Colombia for several reasons, paramount among them is the fact that exposure to indoor radon, both residential and occupational, has not been previously studied. In the context of Central America and the Caribbean, this was the fifth study regarding the number of radon measurements in homes (*n* = 202) [37,38,39,40].

It is well known that the structural and mineralogical characteristics of the ground and underground play a main role in determining the levels of radon emissions from uranium as precursor [41]. Although rocks with relevant uranium concentrations on the Earth’s surface are mostly granitic, basaltic, and sandstones [42] and they are present in some areas in Manizales, exposure to residential ^222^Rn is low in this region according to WHO’s reference levels (100 Bq/m^3^, ideally), given that the highest concentration found in this study was 50 Bq/m^3^.

One factor that could contribute to these low values could be that the area subject to our study, Manizales, is situated close to the Nevado del Ruíz volcano, an active volcano. This volcano erupted in 1985, with abundant debris material expelled (metamorphic rocks and slate). Although the resulting lahars destroyed bridges and homes but did not reach Manizales [43], the explosion’s ashes fallout covered an area of 400 km^2^, and finer ash reached distances 550 km away from the volcano [44]. There are several active volcanos in this area, and it is possible that there may have been other eruptions with exploding ash clouds in the last 2–3 centuries. Nevertheless, there are no registries, and this information cannot be checked. Volcanic ash layers have been acknowledged in some studies to considerably reduce the exhalation of radon from underlying rock to the surface [45,46]. However, the association between volcanic areas and radon is not well established. A paradigmatic example is that of the Canary Islands in Spain, where the two central islands, the most populated, show high levels of radon while radon levels are also very low in the remaining islands in the archipelago [45]. Furthermore, in Hawaii, radon concentrations are also generally low [46].

Another factor that could come into play is the setting of the settlements and, especially, the history of the rocks at the emplacement of Manizales, which have undergone the same evolution as the central mountain range [47], and, to a lesser extent, the fact that it is situated among a chain of mountains, with all that this entails, i.e., winds, mountain rain regime, etcetera. Precipitation as rains stands out as the main meteorological factor in tropical climates and influences degasification levels at ground and underground levels. This study’s measurement period, six months, encompassed the dry (between January and March) and rainy (between April and July) seasons, but without discriminating between them. Studies carried out in Canada, where the differences between seasons are evident, suggest that radon concentration levels do not differ significantly between seasons [48]. There could be some role for the elevation of the measured area. Some studies have suggested that radon may decrease with elevation [49], though the highest concentrations are observed often in mountain areas. This is the case of the Rocky Mountains or Appalachians in the US [50].

Climate characteristics were not assessed directly; only air temperature was checked, which was 18.5 °C in average for the period measurements were taken (minimal temperature was 15.2 °C and maximum 23.3 °C), which is related to the possibility of airing dwellings and avoiding the use of air conditioning. Some lack of indoor isolation of the measured dwellings could also play a role in the results observed, mainly due to the fact that the lowest temperatures in winter are, at best, warm.

For Central and South America, among the few studies that boast a residential radon geometric average [28], the highest level found was in Lages Pintadas, in Brazil (358 Bq/m^3^) [51], followed by Lima, Perú, where it was 155.6 Bq/m^3^ [52]. On the other hand, the lowest geometric mean was found in Natal, Brazil (11.7 Bq/m^3^), although that obtained for Manizales is a bit lower. Several other studies have measured residential radon in Central and South America, but these have not included the geometric mean, which is considered the most appropriate when communicating radon concentration levels, as it follows a lognormal distribution. Detailed information on the comparison of all studies measuring indoor radon in South America can be found in Giraldo-Osorio et al. [28].

It is important to highlight that radon was measured at different heights within the dwellings; the majority of measurements were obtained in the second story of the building. Some indoor radon maps include only street level and first floor measurements [53]. Nevertheless, others include samples taken from buildings with different stories, such as the update of the Dutch radon map [54]. Incorporating homes different from ground floor and first floor level could result in lower concentration levels, but we consider that it allows for a better characterization of the percentages of population exposed to high radon levels. Similar approaches have been published elsewhere [34]. We observed quite similar radon concentrations between rural and urban areas. Given that we observed such low concentrations in the study area, probably due to the fact of low exhalation rates of radon from the ground, we should expect little or no differences of radon concentration between them.

The slightly higher concentration of ^222^Rn in street level residences in comparison to higher stories are deemed to be related to the fact that ^222^Rn is the heaviest of inert gases, with a density of 9.7 g/L at 0 °C and 8 times heavier than air [36]. With regard to the building materials used in the different dwellings, no important differences that could influence radon concentration levels were found. Material of floors showed a difference explained because only three dwellings had this material and the estimations of ^222^Rn were inaccurate, with a wide CI 95% between 0.24 to more than 30 Bq/m^3^. This is in agreement with the results obtained in many other studies that detail this possible source as the least important in the majority of cases as it adds at most 15–20% to total indoor radon levels [1,55].

Given that this was a pilot study, it has certain limitations. One is the scarcity of samples collected in rural areas in relation to its surface (407.02 km^2^, 92.08% of the municipality). A way to minimize this disadvantage is to include geology in the radon mapping method [56,57]. Given that convenience sampling was used, results cannot be extrapolated to neighboring cities nor can other studies in this city be dismissed. Nonetheless, this study is highly relevant because the number of measurements taken was high for this region and currently, there is lack of research in this field in Colombia and also in Central and South America [28]. The study presents several strengths, such as having used alpha-track detectors, recommended by international guides for measuring exposure to residential radon [1]; the distribution, placement, and retrieval of the detectors by two of our researchers, which improved the reliability of the information; the follow up to verify the state of detectors and their position where they were initially placed; the scarce loss of detectors; and their processing in an accredited radon laboratory [58,59].

The experience acquired in this study will enable researchers to widen the areas covered when measuring residential radon in the manner of “stage mapping” in the future. In order to reduce the risk of developing lung cancer, the most important factor is to establish whether there are high levels of radon in homes and, if that is the case, to take measures to reduce them [60]; therefore, the greater number of measurements taken, the more accurate will be the results [55]. It is advisable to produce informative materials so that citizens have access to estimated radon concentrations in their municipalities. So as to develop said information in Colombia, it is necessary to first update the indoor radon reference level and determine its concentration in the air in homes and work places, as there is ample scientific evidence that points at radon as a link to lung cancer [12,61,62,63,64].

## 5. Conclusions

No dwelling surpassed the ^222^Rn residential level established by the WHO. The reason why radon concentrations were low could be the geological nature of subjacent rock in Manizales, given its proximity to active volcano Nevado del Ruíz. Nonetheless, it is advisable to verify this hypothesis and thus advance and strengthen this line of research in Manizales, in other municipalities, and in Colombia itself so as to ascertain the level of residential radon exposure for the different geographical areas and particular geographical characteristics and to create radon maps. In the majority of cases, participants did not have any previous knowledge of radon or the possible health risks derived from exposure to it; therefore, there is an urgent need to promote awareness of this gas in Colombia, both among the general population and among health professionals and public actors. It is necessary to create a national radon program and update the national reference level to match international recommendations. This study also points out the need to further study indoor radon distribution in volcanic areas.

## Figures and Tables

**Table 1:** ^222^Rn concentrations in Manizales, according to dwelling characteristics.

Characteristics	*n*	Geometric Mean	CI 95%
Location area			
Rural	32	8.47	6.27–11.46
Urban	164	8.41	7.35–9.61
Measurement site height			
Below ground	13	7.66	3.67–15.99
Street level	58	10.41	8.36–12.98
First floor	85	7.77	6.56–9.20
Second floor	21	8.16	5.14–12.95
Third floor	6	7.14	2.36–21.60
Fourth floor or more	13	6.88	4.70–10.07
Wall material			
Bahareque/Wood	16	6.31	4.02–9.90
Cement/Fiber cement/Mixed ^1^	52	8.44	6.74–10.57
Brick	128	8.72	7.47–10.17
Material of the floors			
Covered cement ^2^	165	8.86	7.75–10.11
Wood	28	7.04	5.24–9.47
Marble	3	2.76	0.24–31.14

^1^ Mixed: brick and fiber cement; brick and concrete. ^2^ Covering: laminate flooring, floating wood floors, tile, ceramic, porcelain, vinyl, carpet.

## Data Availability

The data presented in this study are available on request from the corresponding author. The data are not publicly available due to confidentiality commitments with study participants.
